# Challenges for the prevention of hypertension among international migrants in Latin America: prioritizing the health of migrants in healthcare systems

**DOI:** 10.3389/fpubh.2023.1125090

**Published:** 2024-01-11

**Authors:** Isabel Rada, Baltica Cabieses

**Affiliations:** ^1^Centro de Salud Global Intercultural (CeSGI), Facultad de Medicina Clínica Alemana, Facultad de Psicología, Universidad del Desarrollo, Santiago, Chile; ^2^Programa de Doctorado en Ciencias e Innovación en Medicina, Instituto de Ciencias e Innovación en Medicina (ICIM), Facultad de Medicina Clínica Alemana, Universidad del Desarrollo, Santiago, Chile; ^3^Department of Health Sciences, University of York, York, United Kingdom

**Keywords:** hypertension, migrants, prevention, health systems, Latin America

## Abstract

Among the health priorities of international migrants, non-communicable diseases such as hypertension are of major interest due to their increasing prevalence, mainly in low- and middle-income countries. Previous evidence has reported a significant risk of hypertension in international migrants derived from multiple exposures during the migration process and at the destination, such as living conditions, health literacy and access to preventive services. Also, poorer disease control has been found compared to the local population. Considering existing deficiencies in access and use of healthcare services related to hypertension prevention and continuity of care of migrants globally, we aimed to offer a Latin American perspective of the challenges faced by international migrants residing in Latin America in accessing hypertension preventive care from a human rights, equity, and universal primary healthcare approaches. From a health systems perspective, we conducted a scoping review of scientific literature on hypertension prevention and control among international migrants in Latin America and the Caribbean. Based on the findings, we discuss the potential influence of migration and health policies on healthcare systems and individual and structural barriers to healthcare access, including lack of insurance, linguistic barriers, limited intercultural competence, and geographical and financial barriers. From existing evidence related to hypertension, we highlight the particular healthcare needs of migrants and their implications for regional public health goals. This aligns with promoting culturally tailored interventions considering the migration process, lifestyle patterns, structural vulnerabilities, and gender particularities in hypertension prevention, diagnosis, and treatment. We advocate for developing universal, voluntary, and systemic regional screening and disease control initiatives in Latin America for hypertension and other chronic conditions.

## 1 Introduction

International migration has been defined as “the movement of people away from their usual residence and across an international border to a country of which they are not nationals” (1). The current worldwide estimation reached 281 million international migrants, representing 3.6% of the world population. Specifically in Latin America, multiple factors have been driving international migration, such as economic and political crises, climate disasters, global and historical social inequalities, violence, and others (2). According to the latest data, of all migrants in the Americas, 58.7 million migrants are residing in North America, and 14.7 million migrants are living in the Latin American and Caribbean region (1.6 million in the Caribbean; 2.3 million in Central America and 10.9 in South America) (3). In recent decades, the migration flows in the South American cone have been mainly intraregional, following a south–south pattern, where 79% of Latin American international migrants reside in another country of the region (2). During the last decade, the historical migration fluxes have changed as sending countries have increasingly become leading destinations (4). Current reports reveal a predominant migration pattern between neighboring countries, possibly due to geographical closeness and, in some cases, multilateral treaties favoring mobility between them (5). In the South American subregion, migrants´ most frequent countries of origin are Venezuela (7.1 million people move from Venezuela, and 85% of them have stayed in the region) (6) followed by Colombia, Paraguay, Bolivia, and Peru. Colombia, Chile, and Peru became the central receiving countries. The main countries of origin of migrants residing in the Central America subregion are EE.UU., Nicaragua, Venezuela, Colombia and Guatemala. Central America’s main receiving countries, including Costa Rica, Panama, Guatemala, Belize and Salvador, represent a corridor for labor migrants transiting to North America (2, 7). Furthermore, mobility restrictions during COVID-19 have impacted migratory flows and increased vulnerability and risks of people aiming to cross during closed borders, with reported adverse effects on mental and physical well-being (8).

Destination countries in the Latin American region have updated their migration policies, such as Chile (Law n° 21.325 of 202), Colombia (Law n° 1.465 of 2011), Peru (Legislative Decree No. 1350 of 2017), Costa Rica (General law of migration n° 8,764 of 2009) Argentina (Migration law n° 25.871 of 2003) and Mexico (Migration Law of 2017). Some of these policies consider access to healthcare for migrants, regardless of administrative status, based on offering equal treatment to nationals and the promise of human rights protection (9, 10). Although the guarantee of equal access is not always explicit in these policies, they promote social security, social integration and the guarantee of emergency medical care at no cost when needed. Some countries like Chile, Mexico, and Peru have explicitly indicated that irregular-status migrants can access healthcare. Migrants in transit stations in Mexico are also entitled to medical attention. Similar protective actions in other countries of the region remain scarce. Noteworthy, migrants face a wide range of challenges and difficulties during the effective implementation of these policies that need further understanding and evaluation (9).

The health of international migrants is considered a global health priority. Hence, recognizing their diversity, exposures, and specific health needs is vital for health planning and service delivery from an equity approach (11). Specifically, non-communicable diseases (NCDs) are major public health concerns among general and migrant populations, especially in destination countries (12). These conditions cause 41 million deaths per year worldwide, corresponding to 71% of all deaths in the general population. NCDs cause premature deaths (30–69 years of age), mainly in low- and middle-income countries (13). They result from genetic predisposition and cumulative risk factors throughout the life course (14). Some evidence suggests that the migratory process might increase NCDs risk in some communities when there is exposure from the country of origin and onwards, depending on the characteristics of living, socio-political, cultural and healthcare conditions. Meanwhile, marginalization, poverty, limited access to secure housing and healthy lifestyles, as well as lack of continuity in care during transit, might exacerbate susceptibility to NCDs. This is coupled with limited access to healthcare and behavioral changes in diet and alcohol and tobacco consumption in the destination country, which gives rise to unique health needs and risks to these populations (15). Late diagnosis and treatment for type II diabetes mellitus, hypertension and cancer have also been reported among international migrants in Latin America and globally, besides poor control of metabolic risk factors for cardiovascular diseases, which can be more prevalent in some migrant communities (13).

Hypertension is one of the most critical risk factors for cardiovascular disease, chronic kidney disease, and premature death. This condition is rapidly increasing in low and middle-income countries due to population aging, urbanization and health-risk behaviors, including unhealthy eating patterns, sedentary lifestyles, and alcohol and tobacco consumption (16). Such individual-risk behaviors are also influenced by structural, persistent, and historical inequalities across and within societies. Globally, it is estimated that there are 1.28 billion adults with hypertension (17), but only 47% of them are under treatment and less than half of them achieve adequate adherence to treatment and disease control (18). The 2030 agenda for sustainable development goals (SDG), through target 3.4, seeks to reduce premature deaths attributable to chronic diseases, along with other targets focused on controlling risk factors and universal health coverage (19). Specific measures to reduce the chronic disease burden include prevention, early diagnosis, and timely treatment (20). In turn, blood pressure control and monitoring in the general population indicates universal health coverage (21).

Migrants are a target group for such global public health goals, as hypertension has become highly prevalent in some migrant communities. In this sense, evidence reports lower disease control rates among migrants compared to local populations (22, 23) and lower awareness of hypertension, which might be influenced by different factors, including barriers to accessing healthcare information and education (13, 24). Health insurance availability also determines awareness, treatment and control of hypertension, being these health-related outcomes lower in those migrants who remain uninsured (25). Access to healthcare is often influenced by migratory policies in the destination country (26). For instance, health disparities between locals and migrants have been reported depending on migratory status; that is, increased cardiovascular risk due to limited access to preventive measures in migrants has been reported in the past (27).

Moreover, continuity of care for NCDs is also influenced by migratory status and migrants’ characteristics. NCDs are not merely affected by access but also to adherence and treatment response considering genetic factors, tailored stratification risk and cultural background (15, 28). Little has been discussed on the increasing hypertension burden among international migrants residing in Latin America, where glaring shortcomings in prevention should be an active part of the regional agenda. The present work aims to offer a regional evidence-based perspective of the growing challenges faced by international migrants in Latin America in accessing hypertension preventive care from human rights, equity and universal primary health care approaches.

## 2 Review methodology

We carried out a scoping review of scientific literature. We aimed for a broad consideration of all papers explicitly addressing any aspect related to Hypertension among international migrants in Latin America and the Caribbean, including refugees. We followed a search in PubMed and Google Scholar with a list of both MeSH and string terms related to “health care OR health systems,” “international migration,” “hypertension,” and “Latin America and the Caribbean” in November 2022 [PubMed = 11 hits / Google Scholar = 152 hits]. Filters were applied to exclude articles including non-human subjects and papers older than ten years. Articles were also identified through searches of the authors’ files. The final reference list was generated based on the following inclusion criteria: (i) Focus on migration and hypertension in Latin America, (ii) Published in the last 10 years, (iii) Available in English, Spanish or Portuguese. Exclusion criteria: non-human migration topics. Finally, we included 53 relevant manuscripts in this manuscript. It is relevant to note that some selected articles were from outside the Latin American region; we decided to include them in this analysis every time they were focused on Latin migrants and barriers to healthcare for hypertension prevention and control. Given that we found limited regional evidence, the inclusion of this type of evidence expanded our capacity to describe and discuss this relevant public health issue. Qualitative thematic data analysis allowed for main common themes to emerge from the existing literature. Our analysis aimed for describing healthcare systems and barriers to health care for hypertension prevention and control among migrants in the region.

## 3 Results

### 3.1 Hypertension among migrants residing in Latin America

According to recent evidence from Colombia, Peru, Chile, Argentina and Uruguay, hypertension affected over 40% of the general population, as indicated by a cross-sectional study of 18 countries in Latin America and the Caribbean. Meanwhile, data from Venezuela revealed a prevalence of 60.4%, and most cases were not under control which may have important implications for those migrating and health systems in destination countries. In addition evidence from afro-descendants in the region is scarce, and underdiagnosis remains a challenge (29). However, data from Haiti reported an age adjusted prevalence of 28.5% and early occurrence compared to other countries (18–30 years) (30). Control of hypertension remained low among the general population in Latin America and the Caribbean region (over 35% of women and 23% of men with hypertension) and little awareness of the disease has been reported, mainly among the uninsured asymptomatic population.

The literature describes the complexity of this public health issue mainly from reports of Latin American migrants residing in North America. For instance, data from Mexican migrants living in the United States revealed a significant impact of systolic hypertension and pre-hypertension on cardiovascular-related deaths, in addition to lower rates of treatment and control compared to locals (26). In Chile, migrants from Haiti self-reported a low prevalence of chronic diseases and hypertension was below 10% rate. In Haiti, albeit country-level data is unknown, it has been suggested that hypertension, preeclampsia and renal disease further affect Haitian migrants due to their African ancestry and social vulnerability (31). Despite the relevance of these studies, evidence of hypertension prevalence of migrants residing in the Latin American region remains scarce and geographically limited (32).

Similar evidence has been reported in South America. In Colombia self-reported prevalence of hypertension in 229 returning Colombians and Venezuelan migrants reached 12.5% (33). Local primary healthcare data analysis confirmed that essential hypertension was a common cause for seeking medical attention in some migrant communities in the region (34). Unique data from the Colombian Ministry of Health and Social Protection reported 9.938 Venezuelan migrants with hypertension out of 16.812 migrants registered with NCDs (35). A population-based study in Peru revealed a prevalence of 2.5% of hypertension in migrants, representing the second most common chronic disease among Venezuelan migrants in that country (36). Evidence from Ecuador described hypertension diagnosis in young adult Latin migrants in either stage I or II during their first year of arrival (37).

Regarding literature from Central America, analyzes of the National Health Survey of Costa Rica showed a self-reported prevalence of 5.7% of hypertension in migrants, with self-reported risk factors including obesity, tobacco consumption and sedentarism (38). Among 392 Mexicans returning to the border of their country of origin, there was a self-reported prevalence of 4.1% diagnosed previous to return (39). According to this data, self-reported prevalence of hypertension in migrants is lower than the reported prevalence in the general population in this region (40%) (29) and worldwide (32%) (18). This is in accordance with previous evidence describing a phenomenon called “healthy migrant effect” (HME) in which migrants tend to display better health outcomes than the local populations (40). Specifically, this health advantage has been described among migrants from South America, Afro-Caribbean countries and Mexico living in EE. UU, whose prevalence were lower than the native-born populations (41–43). Explanatory models describe a possible positive self-selection favoring migration among healthier and younger people, as well as those better skilled and better resourced. Other explanations are focused on previous healthy habits held during migration process and the protective role of psychosocial resources (44, 45). However, this phenomenon can also result from under-registration of the condition in these populations due to lower access to healthcare and their return to the country of origin when feeling sick (46). The HME is particularly relevant in recent migrants and in migrants experiencing socioeconomic and political exclusion and deprivation. Migrants with longer time of residence in the receiving country and those who have been experiencing diverse risks related to marginalization, irregular migration status, socioeconomic deprivation, stigma and discrimination and xenophobia during migration and at arrival, tend to lose their health advantage over time (47).

Based on our review, diverse efforts have been made to address migrant’s health needs in Latin America, including chronic diseases, as evidenced in Chile with the “AUGE-GES” (Explicit Health Guarantees plan) that prioritizes prevalent conditions from which migrants can be benefited regardless insurance status. These efforts conducted by the health system show progress in the region, but like other legal provisions, it should be monitored and reinforced (48). Challenges related to accessing care in migrant populations have been described, which could be exacerbated by administrative and bureaucratic barriers, discrimination within healthcare settings and lack of intercultural pertinence and competence (49). Even in countries that promote access to healthcare for migrants, many Latin American countries show restrictions in healthcare service provision to these populations due to both policy gaps and implementation pitfalls (50, 51). Consequently, experiencing delays and restrictions for healthcare provision might have an impact on the search for private care with out-of-pocket expenditure and the search for treatment outside the healthcare system (48, 52, 53). In this context, there are a few examples -like one from Colombia- in which health policies advocate for health workers training to develop intercultural competences to promote the prevention and early diagnosis of chronic diseases among migrants (51). For example, the “response plan for migrant’s health” in Colombia prioritizes health care attention for diseases that are commonly decompensated during migration like diabetes and hypertension (54).

Evidence suggests a possible relationship between NCDs and migration mediated by the socio-political determination of the health of migrants and refugees (55). This relationship could be catalyzed by historical, pervasive and, in many cases, colonial conditions shaping the individual’s life cycle through economic, political and social unequal power systems (56). Blood pressure is one of the health parameters that is early in life influenced by the socio-political context of living and of migration. In this sense, a high prevalence of NCDs such as hypertension is determined by diverse and multilayered individual and structural risk factors, including gender, age, ethnic background, socioeconomic status, living and working conditions, individual health-related modifiable risks factors (diet, alcohol, tobacco, sedentarism, etc.) and access to healthcare, among others (28). Particularly, evidence suggests that adopting risky lifestyles of the destination country and acculturative stress might impact increased blood pressure (26). At the same time, some migrant groups face greater hypertension prevalence derived from poverty, irregular status, and low level of knowledge on prevention and time of residence, mainly in countries with a high burden of chronic diseases (13, 57).

It is important to make notice of available evidence published during the recent COVID-19 pandemic. During this time, the impact of chronic comorbidities became more visible among migrants in the region. In Mexico, data indicated that hypertension and other cardiovascular risk factors had worsened during transit and at the destination as timely and tailored care was often not available (58). This data suggests that efforts from the healthcare systems in origin, transit and destination countries systems might remain insufficient. Prioritizing this growing public health concern should not rely only on comparisons with local population or prevalence -that is often underreported among migrant communities-, but rather consider migratory fluxes, composition, and exposures of these migrant groups during all stages of the migration process and while residing in host countries.

### 3.2 Health system challenges for equitable preventive services for migrants in Latin America and the Caribbean

Health systems in Latin America and the Caribbean face historical challenges to provide equitable access to everyone while suffering from shortage of resources and system disarticulation (59). Countries in the region are characterized by diverse and heterogeneous financial sources, guarantees of access to care and providers´ performance (60). Most Latin American systems include public subsystems for those in lower socioeconomic strata, as well as social insurance and private subsystems for formal employees and individuals with payment capacity, respectively (61). Many of them also offer complementary private insurance for individuals with higher income (60). A unified public healthcare model has been developed in Cuba and Costa Rica and a public contract model in Brazil. Conversely, most other Latin American countries in the region offer access to healthcare based on a segmented model, depending on the capacity to pay (62).

Health reforms have been proposed towards universal health systems and comprehensive primary health care in countries such as Brazil, Venezuela, Uruguay, and Bolivia, based on State’s responsibility of ensuring the right to health. However, its implementation has been hindered by government changes, low public spending of total health spending and economic crises (60). Thus, these health systems are fragmented and segmented, like those in Chile and Colombia. However, the Chilean and Colombian healthcare systems are market-driven; hence, they have been gradually privatized and increased the cost of care (60). Even though valuable efforts have been progressively made to foster health coverage in Chile (Explicit Health Guarantees), Brazil (Basic Action Plan), Mexico (Popular Security) and Uruguay (National Integrated Health System) (59), the health spending in the region remains low reaching $182/habitant and 3.7% of GDP. This leads to higher direct expenditures on individuals (63) and affects effective access to healthcare services.

When connecting health systems design and performance to hypertension in migrant populations in the region, evidence indicated that actions must be following the individual’s needs, risks and exposures to provide a timely response for informed decision-making (64). To counteract hypertension in the region, initiatives such as the model Hearts in the Americas led by the Pan American Health Organization (PAHO), which supports and fosters cardiovascular disease prevention and its risk factors in collaboration with the Ministries of Health, have been developed (65). This technical package follows the World Health Organization (WHO) guidelines and comprises healthy lifestyle counseling, hypertension treatment and cardiovascular risk assessments while reducing specific care gaps. The initiative is being implemented in 26 countries of the region, including Mexico, Costa Rica, Colombia, Peru, Chile, Argentina, and Brazil, and has improved hypertension control. However, underfunding and pilot implementation limitations remain in some countries, and specific consideration of migrant communities needs further development (65).

Access to health systems among international migrants for hypertension prevention and control in the region entails challenges derived from the disarticulation between migration policies and public health policies. It also needs to address structural, persistent, and historical social, political, and economic disadvantages experienced by marginalized communities in these societies, including some groups of migrants who live in exclusion, poverty, irregularity and multiple forms of stigma and discrimination. Following an intersectional approach, migrant women and those identified with gender diversities, as well as those with a minority ethnic background, concentrate experiences of socioeconomic and political vulnerability and marginalization that affect their health over time. Despite the availability of healthcare services in countries in the region, migrants may have difficulty reaching them. Notably, during their migration process, there are dynamic and multidimensional interactions between the supply of preventive services, level of inclusion in the health system, physical and financial accessibility, information availability, acceptability of services and quality of care sensitive to migration status and their specific needs. Moreover, the demand for preventive services might be influenced by the migrant’s epidemiological profile, beliefs and awareness of health risks, health-seeking behavior, previous exclusion experiences and political and social context of the origin, transit and destination countries (66).

### 3.3 Barriers to accessing hypertension healthcare services among migrants in the region

Available evidence in the region indicates that international migrants are not fully benefited from preventive services. They face critical challenges related to their differentiated health patterns, the need for tailored stratification risk and disease tools, language and cultural barriers and poor knowledge of health care services (67). In addition, healthcare access and use are often limited, particularly among recent and undocumented migrants, either for being ineligible or excluded from health programs and experiencing fear of legal consequences when seeking care (28, 57). However, humanitarian organizations have played a key role in offering complementary responses to the health needs of settled migrants and those in transit throughout Latin America and the Caribbean. For instance, evidence from Colombia described strategies from a primary care approach implemented when access is not guaranteed by migratory status or geographical barriers. This followed the principles for migrants´ comprehensive health care of the Council of Health Ministers of Central America. The medical attention of this model provides preventive services and continuity of care tailored for migrants, either in institutional locations or mobile services located according to the migratory fluxes. The initiative considers the difficulties in treatment adherence faced during the migration process. Thus, permanent medication delivery, monitoring and habit counseling are provided (68).

In Latin American countries, barriers impact the whole population but might be exacerbated for migrants. For instance, health system issues such as lack of coordination and financial prioritization to provide health services and low awareness of migrant’s health rights could leave international migrants behind. Barriers to access to health care involve structural and individual walls that intersect with barriers to health care utilization and social determinants of health (57, 69). Previous evidence from Mexico, Costa Rica, Chile and Colombia has described diverse interactions with healthcare service provision, including migratory status and health policy gaps (51), poor knowledge of health rights and available services, as well as mistrust in medical institutions, low health literacy, not having a regular medical provider, among others. All these factors might hinder access to hypertension preventive services in migrant populations in Latin America (69).

From a regional perspective, and considering the current intraregional migratory fluxes, the absence of coordinated preventive services and continuity of care by health systems of neighboring countries could lead to duplicated efforts, disorientation when navigating different health systems, and increased health risk during transit.

Evidence from preventive services utilization suggested a relationship between discrimination and screening for hypertension; that is, afro-descendant migrants reported experiencing increased levels of discrimination, which limited their use of preventive services (32). Discrimination against migrants could be derived from multiple factors, such as cultural differences, stigma towards a particular ethnicity and language proficiency (26). This issue might decisively affect some migrant communities like Afro-Caribbean and Latin-American Afro-descendant migrants, which might be at higher risk of the disease and lack early prevention. Thus, increasing future projections of disease burden among these vulnerable groups. In addition, a lack of knowledge and misconceptions about the disease and its cost would restrict seeking and reaching appropriate care (26).

On the other hand, in response to diverse barriers, some migrants seek health care in their country of origin either as a substitute or supplement to health services; some have even returned temporarily or permanently for cardiovascular disease treatment (26). The lack of preventive actions aimed at migrants poses severe challenges for public health since hypertension is often asymptomatic and causes major cardiovascular events. However, timely diagnosis and comprehensive treatment would reduce the migratory population’s poor control and disease burden. Inequities in access to preventive medicine and quality health services reveal the selective nature of health coverage instead of universal health coverage throughout the region.

## 4 Discussion

From a health systems perspective, we conducted a scoping review of scientific literature on hypertension prevention and control among international migrants in Latin America and the Caribbean. Our review indicated that international migrants face diverse challenges in their migration process, limiting their access to preventive services. Literature reported the impact of the structure and functioning of health systems at origin, transit, and destination, in addition to the complex and multidimensional interaction of knowledge of health rights, health system and available services, migratory status, immigration and health policies, as well as financial, cultural and language barriers as the most significant determinants of hypertension prevention and control in migrants in the region. These factors act separately and together, producing inequities in access to preventive services and increasing migrant vulnerability (70). Equitable access to quality health services is proposed without financial hardship or discrimination within the framework of universal health coverage and initiatives towards leaving no one behind (71, 72). Access to the health system increases the probability of using preventive services and reducing cardiovascular risk due to access to early screening, diagnosis and timely treatment (43, 73). Although insurance facilitates managing risk factors, lifestyle or dietary changes, it is necessary to optimize access to screening sources and advice on risk factors at first contact with the health systems (73).

Prevention involves diverse efforts across migrant life spans and stages of their migration process. Among the hypertension preventive methods, some engage structural factors to reduce salt intake and increase accessibility to fruits and vegetables, which could benefit the migrant population. However, specific initiatives to ensure universal health coverage and expand primary care might allow more significant contact with health services, large-scale screening programs, and low cost treatment (18). Given the persistent barriers to accessing preventive services and the lack of tailored interventions based on cultural, occupational, accessibility and contextual factors (57), it is expected that poor awareness and control of hypertension in migrants residing in Latin America. Notably, the COVID-19 pandemic makes visible neglected chronic diseases in vulnerable populations along with the effects of vertical health systems, level fragmentation and selective exclusion. Although intersectoral actions of migration experts, civil society engagement and regional agreements to protect health rights have been proposed, it is still pending to reinforce NCDs prevention on the regional agenda.

In understanding health system challenges for equitable access and the growing concern of migrant hypertension risk, poor awareness and control, we call for the design, implementation and evaluation of evidence-based preventive services in Latin America considering the sociosanitary impact of the COVID-19 pandemic. Among migrant groups, some are particularly vulnerable, including those with irregular status, low socioeconomic status and limited access to services. Thus, detecting health care needs and hypertension drivers of diverse migrant groups is critical. Determining health risks upon arrival at transit and destination countries must be regionally coordinated and addressed with intercultural competence. These services may include community-based behavioral interventions adapted to cultural background, gender roles, occupational demands, epidemiological situations and contextual influence on hypertension risk factors (57). In addition, the information provided must facilitate communication and improve knowledge of health rights, the country’s health system and available services to empower migrant groups (26). The latter requires training of health care teams to provide migrant-sensitive services and risk factors management of people on the move.

Gender disparities in hypertension prevalence (e.g., higher in men vs. pre-menopausal women), disease awareness, socioeconomic status, lifestyles and health-seeking behaviors require differential approaches (57, 74). Similarly, there are critical ethnicity-related distinctions in prevalence, control and mortality (e.g., higher prevalence in Afro-descendant migrants), making specific risk stratification tools imperative for diverse populations (28). Therefore, preventive actions should be by gender, ethnicity, acculturation level and NCD profiles of migrants residing in Latin America. Additionally, children and adolescent migrants are susceptible to developing risk factors resulting from early exposure to the migration process and lifestyles of the host society. For instance, evidence suggested the association of migration at earlier ages with cardiovascular risk, creating the need for early prevention focused on these groups (26).

This is, to the best of our knowledge, the first scoping review exploring from a health systems perspective, barriers to accessing effective preventive and control services for hypertension among migrant communities in Latin America and the Caribbean. As a study limitation, some selected articles were from outside the Latin American region, but we decided to include them in this analysis every time they were focused on Latin migrants and barriers to healthcare for hypertension prevention and control. Given that we found limited regional evidence, the inclusion of this type of evidence expanded our capacity to describe and discuss this relevant public health issue. As our main conclusion, a regional initiative of universal, voluntary, and systematic preventive services for hypertension and other chronic diseases should agree with local policies. At the same time, policy adequacy is needed towards migrant-inclusive approaches, promote universal health coverage and reduce disparities to prevent further cardiovascular events (27). Data registry disaggregated by migratory status, gender, and ethnicity, among others, is suggested. To comprehensively study hypertension trends and disparities leading to tailored preventive programs. Furthermore, developing interoperable and secure regional surveillance platforms might help support decision-making. These efforts may implement intersectoral approaches, including members of the migrant and host community, health providers, labor, transport and other relevant sectors for public health benefit (26).

## Data availability statement

The original contributions presented in the study are included in the article/supplementary material, further inquiries can be directed to the corresponding author.

## Author contributions

IR and BC contributed to the conceptualization, synthesis and interpretation of literature, IR drafted the manuscript and BC critically reviewed the manuscript and wrote the final version. All authors contributed to the article and approved the submitted version.
